# Prevalence of High-Risk Human Papillomavirus Infection and Genitourinary Co-Infections with *Chlamydia trachomatis*, *Ureaplasma urealyticum*, *Ureaplasma parvum*, and *Mycoplasma genitalium* in Sexually Active Adolescent Females: A Cross-Sectional Study

**DOI:** 10.3390/jcm15135209

**Published:** 2026-07-03

**Authors:** Mariola Krzyscin, Adam Przepiera, Piotr Łagunowicz, Dominika Pietrzyk, Katarzyna Zając, Alicja Sokołowska, Agnieszka Brodowska, Elżbieta Sowińska-Przepiera

**Affiliations:** 1 Pediatric, Adolescent Gynecology Clinic, Department of Gynecology, Endocrinology and Gynecological Oncology, Pomeranian Medical University in Szczecin, ul. Unii Lubelskiej 1, 71-252 Szczecin, Poland; krzyscin@o2.pl (M.K.); dominikapietrzyk.lote@gmail.com (D.P.); alasok99@gmail.com (A.S.); 2 Department of Urology and Urologic Oncology, Pomeranian Medical University in Szczecin, Powstańców Wielkopolskich 72, 70-111 Szczecin, Poland; 3 Department of Gynecology, Endocrinology and Gynecological Oncology, Pomeranian Medical University in Szczecin, Unii Lubelskiej 1, 71-252 Szczecin, Poland; agnieszka.brodowska@pum.edu.pl; 4 Individual Laboratory for Endocrine Diagnostics, Pomeranian Medical University in Szczecin, 71-252 Szczecin, Poland; elzbieta.sowinska.przepiera@pum.edu.pl

**Keywords:** *Chlamydia trachomatis*, *Ureaplasma urealyticum*, *Mycoplasma genitalium*, HPV, reproductive health, HPV vaccination

## Abstract

**Objectives**: To assess the prevalence of high-risk human papillomavirus (hrHPV) infection and selected genitourinary pathogens and to examine their co-detection patterns in sexually active adolescent females. **Methods**: In this single-center cross-sectional study, 167 consecutive outpatients aged 13–17 years with self-reported sexual initiation underwent multi-pathogen polymerase chain reaction (PCR) testing for hrHPV, *Chlamydia trachomatis* (CT), *Ureaplasma urealyticum* (UU), *Ureaplasma parvum* (UP), and *Mycoplasma genitalium* (MG). Prevalence estimates are reported with 95% confidence intervals (CIs). Associations with hrHPV positivity were explored using univariable and parsimonious multivariable logistic regression; sensitivity analyses examined alternative age parameterization and exclusion of sparse variables. **Results**: The prevalence of hrHPV was 28.1% (47/167; 95% CI: 21.5–35.6). The prevalence of CT, UU, UP, and MG was 3.0%, 28.1%, 25.1%, and 0.6%, respectively. Any bacterial pathogen was detected in 61/167 participants (36.5%), while hrHPV–bacterial co-detection was observed in 24/167 (14.4%). In univariable analysis, UU was associated with hrHPV positivity (OR 2.55, 95% CI: 1.24–5.24; *p* = 0.013); this signal remained in the primary multivariable model (adjusted OR 2.46, 95% CI: 1.07–5.93; *p* = 0.035). A graded increase in hrHPV positivity was observed with increasing bacterial burden (*p* for trend = 0.018). **Conclusions**: Sexually active adolescent girls attending gynecologic outpatient care showed a substantial burden of hrHPV and bacterial genitourinary pathogen detection. This Central and Eastern European adolescent outpatient cohort contributes integrated multi-pathogen PCR-based epidemiologic data from a clinically relevant and underreported population. An exploratory association between UU and hrHPV positivity was observed; this signal is best interpreted as reflecting shared sexual exposure or the cervicovaginal microbial milieu rather than as evidence of an independent causal role for UU. The absence of vaccination status, behavioral, and longitudinal data represents a principal limitation. Prospective studies incorporating these variables are needed to clarify the epidemiology of hrHPV and co-detected pathogens in adolescents.

## 1. Introduction

Human papillomavirus (HPV) infection is the most common sexually transmitted viral infection worldwide. HPV is a common DNA virus that infects epithelial cells in skin and mucous membranes. Cervical, anal, vaginal, and oropharyngeal cancers are strongly linked to high-risk HPV strains like HPV-16 and HPV-18 [1].

Vaccines against HPV are highly immunogenic [2]. Young women who were HPV seronegative prior to immunization have shown exceptionally high levels of vaccine efficacy and effectiveness [3].

Although cervical cancer is largely preventable through HPV vaccination and effective screening strategies, it remains a major global health burden, with approximately 660,000 new cases and 350,000 deaths reported in 2022 [4]. Primary prevention through vaccination constitutes a key pillar of cervical cancer elimination initiatives and is supported by both international and national immunization recommendations. Given the highly contagious and widespread nature of the virus, expanding toward universal vaccination coverage is necessary to maximize population-level protection [5,6].

Adolescents and young women represent a population with high incidence of HPV acquisition shortly after sexual debut. Most HPV infections in this age group are transient; however, a subset persists and may contribute to the development of HPV-related lesions [7]. Factors influencing HPV persistence are multifactorial and may include host susceptibility, sexual exposure patterns, and characteristics of the cervicovaginal microenvironment. In particular, coexisting sexually transmitted infections (STIs) and local inflammatory responses have been hypothesized to affect HPV natural history, potentially facilitating viral acquisition or impairing clearance. Such co-infecting pathogens may create environments conducive to HPV persistence, cellular proliferation, and the acceleration of its oncogenic potential [8,9].

In Poland, suboptimal HPV vaccine uptake and gaps in HPV-related knowledge among adolescents highlight the need for continued strengthening of preventive strategies and improved epidemiological characterization of STI burden in youth. Moreover, data on the co-occurrence of hrHPV with atypical bacterial pathogens such as *Ureaplasma urealyticum*, *Ureaplasma parvum*, and *Mycoplasma genitalium* in sexually active adolescent females remain limited, particularly in Central and Eastern European outpatient populations. This regional evidence gap is relevant because adolescent outpatient cohorts may differ from adult referral populations and from population-based screening samples with respect to exposure timing, vaccination coverage, and referral patterns.

Better characterization of these co-detection patterns may refine adolescent risk stratification, inform counseling in outpatient gynecology, and generate testable hypotheses regarding the interaction between hrHPV and the cervicovaginal microbial environment.

The primary objective of this study was to assess the prevalence of high-risk human papillomavirus (hrHPV) and selected genitourinary infections, as well as their co-occurrence, among sexually active adolescent females. Secondary objectives were to evaluate infection patterns according to chronological age and gynecological age and to explore whether detection of atypical bacterial pathogens was associated with hrHPV positivity. The novelty of the study lies in providing integrated multi-pathogen PCR-based molecular epidemiologic data on hrHPV, CT, UU, UP, and MG in a relatively underreported clinical population of sexually active Polish adolescents from a Central and Eastern European outpatient setting. By focusing specifically on an adolescent gynecologic outpatient cohort rather than an adult or mixed-age population, the study adds clinically grounded epidemiologic detail that is still sparse in the regional literature.

## 2. Materials and Methods

### 2.1. Study Design and Population

This single-center cross-sectional study evaluated the prevalence of high-risk human papillomavirus (hrHPV) infection and selected genitourinary bacterial pathogens among sexually active adolescent females. The study included 167 consecutive participants aged 13–17 years who attended outpatient gynecological or adolescent health services during the study period and for whom complete molecular material was available for hrHPV and bacterial testing. Because the source population was clinic-based rather than population-based, the findings should be interpreted as reflecting infection patterns in sexually active adolescents seeking gynecological care rather than the general adolescent population.

Eligibility criteria were as follows: (1) female sex, (2) age between 13 and 17 years at enrolment, (3) self-reported sexual initiation, and (4) availability of biological specimens suitable for molecular testing of hrHPV and selected bacterial pathogens. Exclusion criteria included recent antimicrobial treatment, known major immunodeficiency, and incomplete laboratory material. A formal prospective screening log was not maintained; however, the analytic cohort was assembled consecutively from eligible outpatients meeting the above criteria.

Data collection was limited to variables predefined in the study protocol and available from medical records or structured clinical interviews. These included chronological age, age at menarche, and laboratory results for hrHPV, CT, UU, UP, and MG. Detailed behavioral variables, including number of sexual partners, condom use, smoking status, and reason for consultation, were not systematically captured and were therefore unavailable for formal adjustment.

Information on HPV vaccination status was not systematically collected as part of the study protocol and was therefore not available for inclusion in the statistical models. This limitation was considered explicitly during interpretation of the multivariable findings.

This manuscript was prepared in accordance with the STROBE recommendations for cross-sectional studies. Particular attention was paid to transparent reporting of participant selection, variable definition, missing data handling, analytic assumptions, and study limitations.

### 2.2. Specimen Collection and Laboratory Procedures

Cervicovaginal swab specimens were collected from all participants by trained healthcare professionals during routine gynecological examinations. Samples were placed in a commercial transport medium and stored under recommended conditions until laboratory processing.

Nucleic acid extraction was performed using standardized protocols in a certified diagnostic laboratory. Detection of high-risk HPV DNA and selected genitourinary pathogens (*Chlamydia trachomatis*, *Ureaplasma urealyticum*, *Ureaplasma parvum*, *Mycoplasma genitalium*) was carried out using polymerase chain reaction (PCR)-based assays validated for clinical use. Cervical swabs were used to collect samples, and they were placed in Universal Transport Medium (UTM™, Copan Diagnostics, Brescia, Italy), which has been shown to preserve HPV DNA and is suggested for use with the GeneProof HPV PCR assay (GeneProof, Brno, Czechia).

Target sequences, amplification conditions, and interpretation criteria followed manufacturer recommendations and established protocols commonly used in epidemiological studies of sexually transmitted infections. Each PCR run included appropriate positive and negative controls to ensure analytical validity. High-risk HPV DNA was detected and partially genotyped using the cobas^®^ HPV test (Roche Diagnostics, Pleasanton, CA, USA), a clinically validated real-time PCR assay targeting the L1 region of the HPV genome. The assay detects 14 high-risk genotypes (HPV-16, -18, -31, -33, -35, -39, -45, -51, -52, -56, -58, -59, -66, and -68), consistent with IARC Group 1 and Group 2A/2B high-risk classification criteria. HPV-16 and HPV-18 are reported individually; the remaining 12 high-risk genotypes are reported as a pooled result. Participants with detection of one or more high-risk genotypes (whether HPV-16, HPV-18, or the pooled channel) were classified as hrHPV-positive; individuals with concurrent positivity for multiple channels were counted as a single hrHPV-positive case in all analyses, and channel-level multiplicity was not examined separately. Detection of bacterial pathogens (CT, UU, UP, and MG) was performed using the Allplex™ STI Essential Assay (Seegene, Seoul, Republic of Korea), a multiplex real-time PCR kit that simultaneously targets species-specific genomic sequences for *Chlamydia trachomatis*, *Ureaplasma urealyticum*, *Ureaplasma parvum*, and *Mycoplasma genitalium*, among other urogenital pathogens. The assay was performed according to the manufacturer’s instructions on a validated real-time PCR platform. Results for each pathogen were recorded as binary (positive/negative) outcomes. These assay details are provided to facilitate methodological comparability with other multi-pathogen PCR studies in similar clinical populations.

### 2.3. Definitions

High-risk HPV infection was defined as PCR detection of one or more HPV genotypes classified as high oncogenic risk according to internationally accepted criteria.

Bacterial infections and HPV–bacterial co-infections were defined as described above.

Chronological age and gynecological age categories were defined a priori, based on biological and developmental considerations.

HPV vaccination status was not included as a study variable and was therefore not considered in the analysis.

Chronological age was categorized into three groups: 13 years, 14–15 years, and 16–17 years, reflecting developmental and behavioral stages typical of adolescence.

Gynecological age (GA) was defined as the number of years elapsed since menarche and was used as a pragmatic proxy for reproductive and cervical maturation. GA was calculated by subtracting age at menarche from age at enrolment and categorized into two groups: 0–4 years and ≥5 years since menarche. This categorization was specified a priori to preserve interpretability and maintain sufficient subgroup counts for stable estimation in a modest-sized adolescent cohort, while acknowledging that some granularity was lost.

### 2.4. Sample Size Justification

The sample size was determined by the number of eligible adolescent girls attending participating outpatient services during the study period who consented to molecular testing and had analyzable specimens. Given previously reported prevalence estimates of hrHPV in adolescent populations, a sample size of 167 allows reasonably precise prevalence estimation but supports only parsimonious multivariable modeling. Accordingly, regression analyses were planned as exploratory association analyses rather than definitive etiologic models.

This sample size is comparable to that used in several cross-sectional outpatient studies of HPV and sexually transmitted infections in adolescents and young women, but it does not eliminate the risk of imprecision for sparse pathogens or small subgroup comparisons.

### 2.5. Statistical Analysis

Statistical analyses were conducted using IBM SPSS Statistics 30 software. The primary outcome was hrHPV positivity, defined as PCR detection of at least one high-risk HPV genotype. Prevalence estimates are presented as counts, percentages, and 95% confidence intervals (CI). Categorical variables were compared using Pearson’s chi-square test or Fisher’s exact test, as appropriate. To provide a more informative descriptive epidemiology, infection patterns were additionally summarized as infection-free, hrHPV only, bacterial infection only, and hrHPV–bacterial co-detection.

Potential sources of bias were considered a priori. Selection bias may have arisen because the cohort was recruited from a specialist outpatient setting. Information bias for the primary exposure and outcome variables was limited because pathogen detection was laboratory-based rather than self-reported. Nevertheless, residual confounding is likely because detailed behavioral variables and HPV vaccination status were unavailable.

Univariable logistic regression analyses were first performed to evaluate crude associations between hrHPV positivity and the presence of *C. trachomatis*, *U. urealyticum*, *U. parvum*, and gynecological age category (0–4 years vs. ≥5 years since menarche). Variables with *p* < 0.20 in univariable analysis, together with clinically relevant covariates defined a priori, were entered into a multivariable logistic regression model.

Given 47 hrHPV-positive events, model complexity was deliberately restricted. The primary multivariable logistic regression model therefore included GA, CT, UU, and UP. MG was analyzed descriptively and in univariable models only, because its prevalence was too low to support stable multivariable estimation. Adjusted odds ratios (aORs) with 95% CIs were calculated. Model fit was assessed using the Hosmer–Lemeshow goodness-of-fit test, Nagelkerke pseudo-R^2^, and the area under the receiver operating characteristic curve (AUC). Multicollinearity was explored using variance inflation factors (VIF). Sensitivity analyses included re-estimation after exclusion of MG and substitution of chronological age categories for GA. A two-sided *p*-value < 0.05 was considered statistically significant. Given the limited number of outcome events relative to the number of candidate predictors, this model should be regarded as exploratory rather than inferentially definitive. Adjusted estimates and confidence intervals should be interpreted with corresponding caution.

Google Gemini (Advanced version, Alphabet Inc., Mountain View, CA, USA) was utilized solely for English language editing, grammatical corrections, and stylistic refinement of the manuscript text.

### 2.6. Laboratory Quality Control

All molecular analyses were conducted in a laboratory operating under standardized quality assurance procedures. Internal quality controls, including positive and negative controls, were incorporated into each PCR run. Laboratory personnel followed standardized operating procedures, and assays were performed using validated commercial kits. Repeat testing was performed in cases of indeterminate or inconclusive results.

### 2.7. Data Handling and Missing Data

Only participants with complete laboratory results for hrHPV and the bacterial pathogens of interest were included in the final analytic dataset. Because the core study variables were laboratory-based, missingness in the final analytic sample was minimal for the primary exposure and outcome variables. No imputation procedures were applied.

### 2.8. Sexual Behavior Variables

Information regarding sexual activity was limited to self-reported sexual initiation, which constituted an inclusion criterion for the study. Detailed behavioral variables such as age at sexual debut, number of sexual partners, condom use, and smoking were not consistently available and were therefore not included in the statistical models. Chronological age and gynecological age were used only as imperfect proxy variables and should not be interpreted as substitutes for direct measures of sexual exposure.

### 2.9. Ethical Considerations

According to the decision of the Bioethics Committee of the Pomeranian Medical University in Szczecin (Ref. No. KB.006.43.2024; 20 March 2024), the submitted study description did not require a formal opinion of the Bioethics Committee. The study was conducted in accordance with the Declaration of Helsinki and institutional standards for the use of anonymized clinical and laboratory data.

All data were analyzed in anonymized form, and only participants with complete laboratory material available for the predefined molecular analyses were included in the final analytic dataset.

## 3. Results

### 3.1. Characteristics of the Study Population

A total of 167 sexually active adolescent females aged 13–17 years were included in the final analytic sample. The cohort represented consecutive outpatients with complete molecular test results for hrHPV, CT, UU, UP, and MG. Because a formal screening log was not maintained prospectively, the exact number of initially assessed patients could not be reconstructed with certainty; however, only participants with complete analyzable laboratory material entered the final dataset. Most participants were aged 16–17 years (*n* = 123, 73.7%), followed by those aged 14–15 years (*n* = 33, 19.8%) and 13 years (*n* = 11, 6.6%).

Based on gynecological age, 122 participants (73.1%) were classified as GA 0–4 years, whereas 45 participants (26.9%) had a gynecological age of 5 years or more. Overall, 83 participants (49.7%) were infection-free with respect to the tested pathogens, 23 (13.8%) had hrHPV without bacterial co-detection, 37 (22.2%) had bacterial pathogen detection without hrHPV, and 24 (14.4%) had concomitant hrHPV and at least one bacterial pathogen.

### 3.2. Prevalence of High-Risk HPV and Genitourinary Infections by Age

Between-group comparison for hrHPV prevalence across chronological age categories: chi-square = 2.22, df = 2, *p* = 0.329. Because several non-HPV pathogen categories contained sparse cells, age-stratified comparisons for the rarest pathogens were interpreted descriptively and Fisher exact testing was considered more appropriate than omnibus chi-square testing where expected counts were <5 (Table 1).

High-risk HPV infection was detected in 47 of 167 participants (28.1%; 95% CI: 21.5–35.6). The prevalence of hrHPV varied across age groups, reaching 36.4% among 13-year-old girls, 18.2% in those aged 14–15 years, and 30.1% in the 16–17-year age group. The overall age-group comparison was not statistically significant (chi-square = 2.22, df = 2, *p* = 0.329), and no monotonic age trend was apparent, likely reflecting the small youngest subgroup and the uneven age distribution of the clinic-based cohort.

Among bacterial pathogens, *Ureaplasma urealyticum* was the most frequently detected microorganism (47/167; 28.1%; 95% CI: 21.5–35.6), followed by *Ureaplasma parvum* (42/167; 25.1%; 95% CI: 18.8–32.4), *Chlamydia trachomatis* (5/167; 3.0%; 95% CI: 1.0–6.9), and *Mycoplasma genitalium* (1/167; 0.6%; 95% CI: 0.0–3.3). Any bacterial pathogen was detected in 61/167 participants (36.5%; 95% CI: 29.2–44.2). Although bacterial detections were numerically concentrated in the 16–17-year subgroup, these descriptive differences should be interpreted cautiously because the age groups were highly unbalanced.

### 3.3. Distribution of Infections According to Gynecological Age

**Figure 1:**
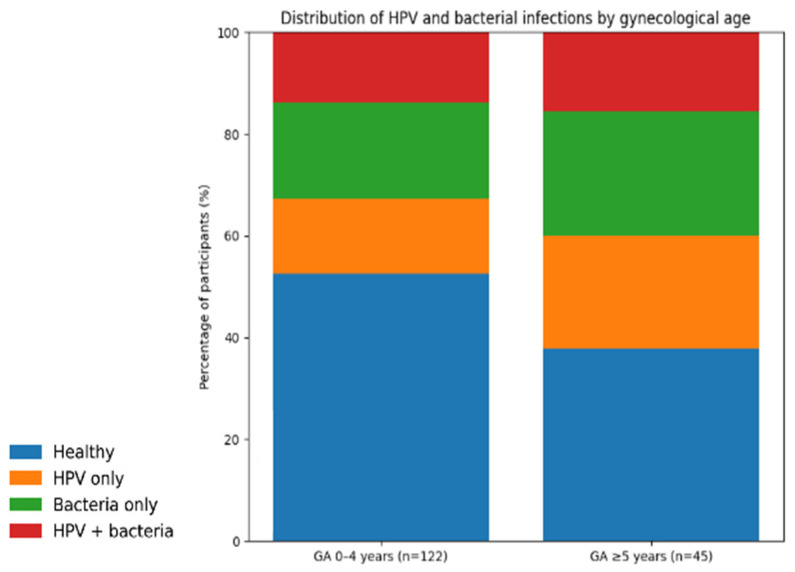
Distribution of infection status according to gynecological age. Categories comprise infection-free participants, hrHPV only, bacterial infection only, and hrHPV–bacterial co-detection. In GA 0–4 years (*n* = 122), 64 girls (52.5%) were infection-free, 18 (14.8%) had hrHPV only, 24 (19.7%) had bacterial detection only, and 16 (13.1%) had hrHPV–bacterial co-detection. In GA ≥ 5 years (*n* = 45), the corresponding counts were 19 (42.2%), 5 (11.1%), 13 (28.9%), and 8 (17.8%), respectively. Overall comparison of infection-status distribution between GA categories: chi-square = 2.77, df = 3, *p* = 0.429.

Among participants with a gynecological age of 0–4 years, just over half were free of detectable infection (64/122; 52.5%). Isolated hrHPV infection was observed in 18 girls (14.8%), isolated bacterial detection in 24 (19.7%), and hrHPV–bacterial co-detection in 16 (13.1%). The distribution of infection status according to gynecological age is shown in Figure 1.

In girls with a gynecological age of 5 years or more, the proportion of infection-free individuals was lower (19/45; 42.2%), while isolated bacterial detection (13/45; 28.9%) and hrHPV–bacterial co-detection (8/45; 17.8%) were numerically more common than in the lower-GA group. Isolated hrHPV without bacterial co-detection was observed in 5/45 girls (11.1%).

Although these distributions suggest a somewhat less favorable infection profile in girls with greater gynecological age, the overall association between GA category and infection pattern was not statistically significant (chi-square = 2.77, df = 3, *p* = 0.429). These findings support interpretation of GA as a useful descriptive stratifier rather than a strong independent determinant within this sample.

### 3.4. HPV and Genitourinary Co-Infections

**Figure 2:**
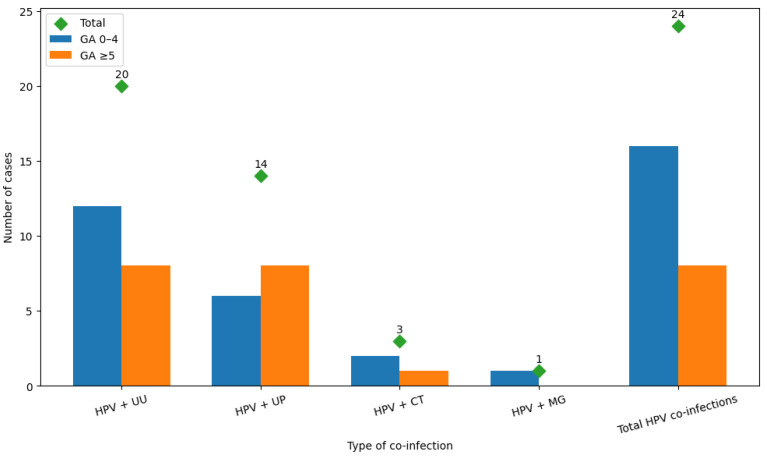
Distribution of high-risk HPV co-infections with selected genitourinary pathogens according to gynecological age Bar chart showing the absolute number of adolescent girls with high-risk human papillomavirus (HPV) infection co-occurring with selected genitourinary pathogens, stratified by gynecological age (GA 0–4 years vs. GA ≥ 5 years). Co-infections include *Ureaplasma urealyticum* (UU), *Ureaplasma parvum* (UP), *Chlamydia trachomatis* (CT), and *Mycoplasma genitalium* (MG). The total number of HPV co-infections across all age groups is indicated. Green markers indicate the total number of cases for each co-infection category. Comparison of any hrHPV–bacterial co-detection between GA categories: chi-square = 0.55, df = 1, *p* = 0.458.

Figure 2 illustrates the bacterial composition of hrHPV co-detection patterns rather than mutually exclusive patient groups. The most frequent hrHPV co-detection involved UU (20/47 hrHPV-positive girls; 42.6%), followed by UP (14/47; 29.8%). Less common co-detections involved CT (3/47; 6.4%) and MG (1/47; 2.1%).

High-risk HPV co-detection with at least one bacterial pathogen was identified in 24 participants, accounting for 14.4% of the total cohort and 51.1% of all hrHPV-positive participants. Any bacterial pathogen was detected more often among hrHPV-positive girls than among hrHPV-negative girls (24/47 [51.1%] vs. 37/120 [30.8%]; OR 2.34, 95% CI: 1.15–4.75; *p* = 0.019).

The distribution of any hrHPV–bacterial co-detection differed numerically by gynecological age, with 16 cases observed among girls with GA 0–4 years and 8 cases among those with GA ≥ 5 years. Given the unequal denominator sizes, these counts corresponded to 13.1% and 17.8% of each GA group, respectively.

Accordingly, no statistically significant association was found between GA category and the presence of any hrHPV–bacterial co-detection (chi-square = 0.55, df = 1, *p* = 0.458).

### 3.5. Factors Associated with High-Risk HPV Infection

Univariable and multivariable logistic regression analyses of factors associated with hrHPV positivity are presented in Table 2. In univariable analysis, UU showed a statistically significant crude association with hrHPV positivity (OR 2.55, 95% CI: 1.24–5.24; *p* = 0.013). CT and greater gynecological age showed directional but non-significant associations, whereas UP was not associated with hrHPV in crude analyses. The very low prevalence of MG precluded meaningful inference beyond descriptive reporting. In interpretive terms, the UU signal may be more appropriately understood as a marker of shared sexual exposure patterns or of the broader cervicovaginal microbial milieu than as evidence of a direct biologic effect in this dataset.

In the primary multivariable logistic regression model, an exploratory association between UU and hrHPV positivity was observed (adjusted OR 2.46, 95% CI: 1.07–5.93; *p* = 0.035). Given the modest number of hrHPV-positive events (*n* = 47) and the exploratory nature of this analysis, these results must be interpreted with caution. The finding should be viewed as a signal of potential microbial co-occurrence rather than evidence of a robust clinical or biological determinant. Additional descriptive and sensitivity analyses of bacterial burden and hrHPV positivity are presented in Table 3.

Trend across bacterial-burden categories—*p* for trend = 0.018. Relative to participants with no bacterial pathogen detected, the odds of hrHPV positivity were higher for those with one detected pathogen (OR 2.00, 95% CI 0.93–4.31) and highest for those with ≥2 pathogens (OR 3.23, 95% CI 1.17–8.90). A graded increase in hrHPV positivity was observed with increasing bacterial burden (*p* for trend = 0.018). This analysis should be interpreted descriptively, because individual bacterial categories were not mutually exclusive with respect to pathogen type and subgroup sizes were modest. Overall, the data from these models illustrate a collaborative pattern of multi-pathogen co-occurrence; while the exact reasons for this collaboration remain to be elucidated, it likely reflects combined behavioral exposures and synergistic alterations in the local microflora.

## 4. Discussion

This cross-sectional study provides molecular epidemiologic data on hrHPV and selected genitourinary pathogens in sexually active adolescent females attending a gynecologic outpatient setting in Poland. Three findings merit emphasis. First, the burden of detectable infection was substantial: hrHPV was present in 28.1% of the cohort, any bacterial pathogen in 36.5%, and hrHPV–bacterial co-detection in 14.4%. Second, UU was the bacterial pathogen most frequently detected overall and the pathogen most often co-detected with hrHPV. Third, an exploratory association between UU positivity and hrHPV positivity was observed in the multivariable model (adjusted OR 2.46, 95% CI: 1.07–5.93; *p*  =  0.035); however, this finding is best interpreted as a potential epidemiological signal of shared sexual exposure or of the broader cervicovaginal microbial milieu, rather than as evidence of a direct, independent causal role for UU in hrHPV infection. Given the cross-sectional design, modest sample size, and absence of key behavioral and vaccination covariates, no causal inference is warranted from this association. Beyond these numerical findings, the study contributes region-specific evidence from Central and Eastern Europe and focuses on a consecutive adolescent outpatient cohort that is less often represented in the literature than adult or mixed-age populations.

These findings should be interpreted primarily as clinically informative and hypothesis-generating rather than mechanistically definitive. The novelty of the present study lies in the integrated multi-pathogen PCR-based assessment of hrHPV together with CT, UU, UP, and MG in an underreported outpatient adolescent population from Central and Eastern Europe. In contrast to studies focused on adults or mixed-age gynecology cohorts, the present analysis targets adolescent girls specifically and examines infection patterns through both chronological age and gynecological age lenses. The use of a combined molecular panel also allows the study to move beyond single-pathogen reporting toward a more clinically realistic description of co-detection patterns in adolescent outpatient care.

Our prevalence estimates are broadly consistent with outpatient-based studies showing frequent co-detection of HPV and bacterial STIs, including Ureaplasma species, although direct comparison is limited by differences in age composition, health-care setting, and diagnostic panels [10,11,12,13,14]. The hrHPV prevalence observed here also falls within the range reported in sexually active, largely non-vaccinated adolescent cohorts, in whom HPV acquisition commonly occurs soon after sexual debut [7,15,16,17].

Beyond their microbiological relevance, the observed prevalence patterns have important public health implications. The higher burden of infections among older adolescents suggests increasing cumulative exposure during late adolescence. The relatively high prevalence of hrHPV and *Ureaplasma* spp. highlights a substantial infectious burden in this population at a key stage of sexual development. These findings underscore the importance of timely preventive measures implemented before or shortly after sexual debut. In particular, they highlight the central role of comprehensive, evidence-based sexual health education as a key pillar of STI prevention, alongside HPV vaccination and risk-reduction counseling. Although sexual health knowledge was not assessed, the results support strengthening early preventive education in adolescents.

The substantial prevalence of these pathogens highlights a critical gap in educational initiatives regarding the risks of early, unprotected sexual intercourse. From a clinical perspective, while our dataset did not capture specific symptoms such as abnormal discharge, itching, or pain, the collaboration of HPV with concomitant STIs is known to exacerbate clinical presentations and increase the risk of recurrent lesions and oncogenic progression. Consequently, a comprehensive diagnostic approach is highly recommended; the detection of hrHPV should prompt screening for other major STIs to ensure concomitant pathogens are identified. Furthermore, counseling must heavily emphasize the use of barrier methods of contraception, which significantly reduce the risk of both initial reinfection and the development of multi-pathogen collaboration.

The observed UU–hrHPV pattern is compatible with prior literature linking genital microecology and bacterial co-detection patterns to HPV positivity [10,14,18,19,20,21]. However, our data do not establish that UU is a causal cofactor of hrHPV acquisition or persistence. Ureaplasma species are common in sexually active populations, and their detection may reflect colonization rather than clinically meaningful infection [22]. Accordingly, UU should be viewed here primarily as a marker of sexual exposure and of the underlying cervicovaginal microbial milieu associated with hrHPV positivity in the available dataset, rather than as proof of an independent etiologic role.

A biologically plausible framework exists for an association between bacterial dysbiosis, local inflammation, and HPV natural history [18,20,21,23,24,25]. Nevertheless, any mechanistic interpretation must remain cautious in a cross-sectional study without longitudinal follow-up, quantitative microbial load data, cervical cytology outcomes, or direct microbiome profiling. The present findings therefore support future prospective work rather than causal claims about persistence or progression. They may, however, help refine the choice of variables to be captured in future adolescent studies, particularly where molecular pathogen detection, vaccination status, and behavioral risk measures can be evaluated jointly.

The lack of a statistically significant independent effect of gynecological age should not be overinterpreted as evidence that maturation is irrelevant to adolescent HPV epidemiology. Rather, GA was used here as a pragmatic developmental proxy. The absence of a strong signal may reflect limited power, broad categorization, and residual confounding by unmeasured behavioral variables such as time since sexual debut [26,27]. Importantly, the sensitivity model using chronological age instead of GA produced a similar UU estimate, which increases confidence that the main association was not solely an artifact of the age parameterization chosen for the primary model.

One of the main strengths of the study is the use of PCR-based testing for all pathogens of interest, which limits misclassification of the primary exposure and outcome variables. Additional strengths include the clinically focused adolescent cohort, the simultaneous assessment of multiple atypical bacterial pathogens, and the presentation of complementary descriptive, univariable, multivariable, and sensitivity analyses. These elements modestly enhance interpretability compared with a purely descriptive prevalence report, while remaining exploratory in nature.

The study also has important limitations. It was single-center and clinic-based, which may limit generalizability and introduce referral-related selection bias. The age distribution was imbalanced toward older adolescents. Most importantly, detailed behavioral and preventive variables—including age at sexual debut, number of sexual partners, condom use, smoking, symptom status, indication for attendance, and HPV vaccination status—were not systematically available. Residual confounding is therefore highly likely, and the observed UU–hrHPV association may partly reflect shared exposure pathways rather than a direct biological effect. In addition, sparse counts for MG and CT limited precision, and the multivariable model, although intentionally parsimonious, should still be regarded as exploratory. These missing variables represent the principal limitation of the present study and are explicitly acknowledged throughout the manuscript. Future studies in this population should prioritise prospective collection of vaccination status, number of sexual partners, age at sexual debut, condom use, and symptom indication for attendance, as these are essential for a robust interpretation of HPV and co-infection epidemiology in adolescents.

From a clinical and public-health perspective, the present data reinforce the importance of comprehensive adolescent sexual health care. These findings do not support routine UU screening or eradication in asymptomatic adolescents, which would be inconsistent with current guidance [22]. Rather, detection of UU in this context is more appropriately interpreted as a possible marker of exposure profile or cervicovaginal microbial milieu, not as a standalone therapeutic target. Clinically, the results support risk-reduction counseling, evidence-based STI testing where indicated, and strengthening HPV vaccination uptake before or soon after sexual debut [5,6]. In Poland, where vaccine uptake remains suboptimal, adolescent outpatient encounters may represent an important opportunity for prevention-oriented counseling and vaccine advocacy [28,29].

## 5. Conclusions

This study demonstrates a substantial burden of hrHPV and bacterial genitourinary pathogen detection in sexually active adolescent girls attending outpatient gynecologic care. The most consistent signal observed in this dataset was an exploratory association between UU positivity and hrHPV detection, which is best interpreted as reflecting shared sexual exposure patterns or the broader cervicovaginal microbial milieu rather than evidence of a direct causal or biological role.

The principal contribution of the study is to provide integrated multi-pathogen PCR-based epidemiologic data on hrHPV, CT, UU, UP, and MG in a relatively underreported Polish adolescent clinical population from Central and Eastern Europe. By combining age-stratified prevalence estimates, infection-pattern analyses, and parsimonious multivariable modeling in a consecutive adolescent outpatient cohort, the study moves beyond a simple prevalence report while remaining transparent about its methodological limits.

From a public health perspective, the findings support strengthening comprehensive sexual health education, risk-reduction counseling, and timely access to evidence-based STI testing where clinically indicated. They also reinforce the need to improve HPV vaccination uptake in adolescents, particularly before or near the onset of sexual activity. At the same time, the results do not justify routine UU screening in asymptomatic adolescents and should not be read as support for organism-directed intervention in the absence of established clinical indications.

Despite the absence of individual vaccination data in this cohort, current recommendations strongly support early HPV immunization as the cornerstone of primary prevention [5,6]. Prospective multicenter studies with richer behavioral, vaccination, and longitudinal follow-up data are needed to clarify whether observed co-detection patterns have implications for hrHPV persistence, clearance, or downstream cervical outcomes.

Taken together, these findings should be regarded as descriptive and exploratory observations from a real-world adolescent outpatient setting. Prospective multicenter studies should include HPV vaccination status, number of sexual partners, age at sexual debut, condom use, and smoking as primary covariates, and should incorporate longitudinal outcome data to clarify whether multi-pathogen co-detection patterns have implications for hrHPV persistence or cervical disease progression.

Implementation of age-appropriate sexual health programs, improved prevention messaging, and stronger integration of STI counseling with HPV vaccination efforts should remain priorities for reducing long-term reproductive health risks among adolescents.

## Figures and Tables

**Table 1:** Prevalence of high-risk HPV and selected genitourinary pathogens stratified by chronological age.

Age Group (Years)	13	14–15	16–17	Total
Number of patients	11	33	123	167
high-risk HPV, *n* (%)	4 (36.4)	6 (18.2)	37 (30.1)	47 (28.1)
*Chlamydia trachomatis*, *n* (%)	0 (0.0)	0 (0.0)	5 (4.1)	5 (3.0)
*Ureaplasma urealyticum*, *n* (%)	0 (0.0)	10 (30.3)	37 (30.1)	47 (28.1)
*Ureaplasma parvum*, *n* (%)	0 (0.0)	6 (18.2)	36 (29.3)	42 (25.1)
*Mycoplasma genitalium*, *n* (%)	0 (0.0)	0 (0.0)	1 (0.8)	1 (0.6)

Values are presented as absolute numbers (*n*) and percentages (%).

**Table 2:** Univariable and primary multivariable logistic regression analyses of factors associated with hrHPV positivity (*n* = 167).

Variable	hrHPV-Positive/Exposed, *n*/*N* (%)	Crude OR (95% CI)	*p*-Value	Adjusted OR (95% CI)	*p*-Value
*C. trachomatis*	3/5 (60.0)	4.02 (0.65–24.89)	0.136	3.40 (0.51–22.50)	0.205
*U. urealyticum*	20/47 (42.6)	2.55 (1.24–5.24)	0.013	2.46 (1.07–5.93)	0.035
*U. parvum*	14/42 (33.3)	1.39 (0.66–2.97)	0.430	0.90 (0.33–2.41)	0.830
GA ≥ 5 years	16/45 (35.6)	1.62 (0.78–3.37)	0.201	1.34 (0.61–2.96)	0.468

OR—odds ratio; CI—confidence interval; GA—gynecological age.

**Table 3:** Additional descriptive and sensitivity analyses of bacterial burden and hrHPV positivity.

Bacterial Burden Category	Total, *n* (%)	hrHPV-Positive, *n*/*N* (%)	Crude OR (95% CI)	*p*-Value
0 bacterial pathogens detected	106 (63.5)	23/106 (21.7)	Reference	—
1 bacterial pathogen detected	42 (25.1)	15/42 (35.7)	2.00 (0.93–4.31)	0.077
≥2 bacterial pathogens detected	19 (11.4)	9/19 (47.4)	3.23 (1.17–8.90)	0.024

## Data Availability

Data are available on special request after contacting author (M.K.).

## Abbreviations

The following abbreviations are used in this manuscript:

PCR	polymerase chain reaction
HPV	Human papillomavirus
CT	*Chlamydia trachomatis*
UU	*Ureaplasma urealyticum*
UP	*Ureaplasma parvum*
MG	*Mycoplasma genitalium*
STIs	Sexually transmitted infections
GA	Gynecological age
